# Hospital Readmissions Among Veterans Within 90 Days of Discharge Following Initial Hospitalization for COVID-19

**DOI:** 10.5888/pcd19.220200

**Published:** 2022-12-01

**Authors:** Frances M. Weaver, Meike Niederhausen, Alex Hickok, Allison C. O’Neill, Howard S. Gordon, Samuel T. Edwards, Diana J. Govier, Jason I. Chen, Rebecca Young, Mary Whooley, Denise M. Hynes

**Affiliations:** 1 Center of Innovation for Complex Chronic Healthcare, Hines VA Hospital, Hines, Illinois; 2 Parkinson School of Health Sciences and Public Health, Loyola University, Maywood, Illinois; 3 Center to Improve Veteran Involvement in Care, Portland VA Hospital, Portland, Oregon; 4 Oregon Health and Science University–Portland State University School of Public Health, Oregon Health and Science University, Portland, Oregon; 5 Jesse Brown Veterans Affairs Medical Center and Veterans Affairs Center of Innovation for Complex Chronic Healthcare, Chicago, Illinois; 6 Section of Academic Internal Medicine, Department of Medicine, University of Illinois at Chicago, Chicago, Illinois; 7 Institute for Health Research and Policy, University of Illinois at Chicago, Chicago, Illinois; 8 Section of General Internal Medicine, Veterans Affairs Portland Healthcare System, Portland, Oregon; 9 Department of Psychiatry, Oregon Health and Science University, Portland, Oregon; 10 San Francisco Veterans Affairs Health Care System, and the University of California, San Francisco, California; 11 Health Management and Policy, School of Social and Behavioral Sciences, College of Public Health and Human Sciences, Oregon State University, Corvallis, Oregon; 12 School of Nursing, Oregon Health and Science University, Portland, Oregon

## Abstract

**Introduction:**

Some patients experience ongoing sequelae after discharge, including rehospitalization; therefore, outcomes following COVID-19 hospitalization are of continued interest. We examined readmissions within 90 days of hospital discharge for veterans hospitalized with COVID-19 during the first 10 months of the pandemic in the US.

**Methods:**

Veterans hospitalized with COVID-19 at a Veterans Health Administration (VA) hospital from March 1, 2020, through December 31, 2020 were followed for 90 days after discharge to determine readmission rates.

**Results:**

Of 20,414 veterans hospitalized with COVID-19 during this time period, 13% (n = 2,643) died in the hospital. Among survivors (n = 17,771), 16% (n = 2,764) were readmitted within 90 days of discharge, with a mean time to readmission of 21.6 days (SD = 21.1). Characteristics of the initial COVID-19 hospitalization associated with readmission included length of stay, mechanical ventilator use, higher comorbidity index score, current smoking, urban residence, discharged against medical advice, and hospitalized from September through December 2020 versus March through August 2020 (all *P* values <.02). Veterans readmitted from September through December 2020 were more often White, lived in a rural or highly rural area, and had shorter initial hospitalizations than veterans hospitalized earlier in the year.

**Conclusion:**

Approximately 1 of 6 veterans discharged alive following a COVID-19 hospitalization from March 1 through December 31, 2020, were readmitted within 90 days. The longer the hospital stay, the greater the likelihood of readmission. Readmissions also were more likely when the initial admission required mechanical ventilation, or when the veteran had multiple comorbidities, smoked, or lived in an urban area. COVID-19 hospitalizations were shorter from September through December 2020, suggesting that hospital over-capacity may have resulted in earlier discharges and increased readmissions. Efforts to monitor and provide support for patients discharged in high bed–capacity situations may help avoid readmissions.

**Summary**

**What is already known on this topic?**

Readmissions following a COVID-19 hospitalization are not uncommon. Most studies have only examined readmission within the first 30 days.

**What is added by this report?**

Our examination of readmissions up to 90 days following discharge confirmed that readmissions following COVID-19 occurred in a short period (median of 14 days) and were related to people who were sickest during their initial hospitalization. Furthermore, when hospitals were overwhelmed, initial hospital length of stay decreased, resulting in an increase in readmission rates.

**What are the implications for public health practice?**

Our findings suggest that post-discharge monitoring and intervention may be key to reducing readmissions.

## Introduction

The SARS-CoV-2 virus has infected more than 91 million people worldwide and resulted in more than 1 million deaths in the US alone as of August 2022. Much has already been published about the acute effects of COVID-19, but less is known about people who survive hospitalization and are discharged, particularly as the pandemic continues over time. An estimated 9% to 40% of people who test positive for COVID-19 are hospitalized, with higher rates among those with underlying health conditions (1). Many people hospitalized endured long and difficult stays involving respiratory failure requiring mechanical ventilation and days — even weeks — in intensive care units (ICUs). Richardson et al (2) reported that of patients hospitalized with COVID-19 across 12 hospitals in New York City from March 1 through April 4, 2020, 14.2% spent time in an ICU and 21% died. Another study found that of 2,179 veterans treated in Veterans Health Administration (VA) hospitals, 31% were treated in an ICU, 14% received mechanical ventilation, and 19% died during hospitalization (3).

The long-term toll from COVID-19 hospitalization among survivors is still being realized. Thirty-day readmissions following a COVID-19 hospitalization across studies and countries have averaged around 8% to 9% (4–6). Lavery and colleagues (7) examined readmission rates for 126,137 patients hospitalized with a COVID-19 diagnosis from March through July 2020. Among those discharged, 60% were discharged to home or self-care without additional health care services, and 15% were discharged to a skilled nursing facility. Nine percent were readmitted within 2 months of their initial discharge. Readmissions were associated with older age (≥65 y), being of a race other than White, being male, having a chronic condition (ie, chronic obstructive pulmonary disease, heart failure, diabetes, chronic kidney disease), having received mechanical ventilation during hospitalization, and being discharged to either home health, a skilled nursing facility, or ongoing care in another acute care facility. Similarly, Rokadiya and colleagues reported a 6% rehospitalization rate for a cohort of 391 COVID-19 patients discharged from a hospital in England (8). Approximately one-quarter of these readmissions resulted in death or discharge into end-of-life care. Another study examined readmissions and deaths following a COVID-19 hospitalization in a national sample of veterans admitted to VA hospitals between March and June 2020 (3). Among the 1,775 veterans discharged alive, 27% were readmitted or died within 60 days. The most common reason for readmission was COVID-19–related (30%). When compared with matched cohorts of hospitalized pneumonia and heart failure patients, COVID-19 patients had lower rates of readmission or death in 60 days, but higher rates within the first 10 days of discharge. Studies that examined longer follow-up of patients discharged after a COVID-19 admission reported readmission rates from 24% to 30% (5,9).

Most of the literature has reported on readmissions during the first few months of the pandemic. Less is known about readmission rates following COVID-19 hospitalizations as the pandemic continued. Furthermore, these early data suggest that veterans hospitalized with COVID-19 in VA hospitals may have experienced higher readmission rates than the general population. Underlying comorbidities and the increasing health system burden may explain some of this difference. The purpose of our study was to examine hospital readmissions among veterans within 90 days of discharge following initial hospitalization for COVID-19 during the 3 initial waves of COVID-19 infection in the first year of the pandemic, and before vaccines were available. We included an expanded discharge period of 90 days to capture possible exacerbations of chronic conditions following a COVID-19 infection in addition to readmissions related to COVID-19 issues.

## Methods

We identified veterans hospitalized in a VA facility with a positive diagnosis of COVID-19 that was based on a positive polymerase chain reaction test indicating the presence of SARS-CoV-2, from March 1, 2020, through December 31, 2020 (N = 20,571). Data sources included the VA COVID-19 Shared Data Resource, which was created to assist researchers and policy makers in identifying and studying veterans who were tested for COVID-19 (10). Electronic health records from the VA Corporate Data Warehouse were also used for identifying hospital admissions, outpatient encounters, and comorbidities and for additional sociodemographic characteristics (11,12). VA hospital readmission for acute care was assessed within 90 days of the initial COVID-19 discharge for those patients who were discharged alive.

Patient sociodemographic measures included those used in our prior work (13,14): race (consolidated from 6 to 3 categories due to low cell counts: White, Black, or other listed races [which included Asian, American Indian/Alaska Native, Native Hawaiian/Pacific Islander, unknown race]); ethnicity (Hispanic/Latino or not); sex (male or female); age in years (calculated from birthdate to date of first COVID-19 admission); and marital status (consolidated from 4 categories to 2, married versus single). VA copayment categories were based on VA priority grouping and copayment levels (none, groups 1–3; some, groups 4–6; or full copay, groups 7 and 8) (15). Insurance status was categorized as only VA coverage, VA and private insurance, VA plus Medicare and/or Medicaid (public), and VA with public plus private insurance. Rural status was based on the veteran’s recorded residence and US census data (urban, rural, highly rural) (16). Discharge locations were to community, another hospital or long-term care, discharged against medical advice, or data missing.

Patient clinical characteristics included were smoking status (never, former, current, or unknown), body mass index (weight in kg/height in m^2^), length of stay (days), and whether a ventilator was required during the initial COVID-19 hospitalization. To ascertain comorbidities, we used the Elixhauser method to score 30 disease-specific conditions and a summary index, calculated by using a 2-year look-back period from hospital admission (17–19). For modeling purposes, the Elixhauser score was scaled down by a factor of 10. Reasons for readmission were based on the Agency for Healthcare Research and Quality’s Clinical Classifications Software for reorganizing and collapsing the International Classification of Diseases codes, developed as part of their Healthcare Cost and Utilization Project (HCUP) (20).

Vital status was confirmed by using the VA Vital Status file, which includes mortality data from the Veterans Administration, Medicare, and the Social Security Administration (21). On the basis of the admission date of their initial COVID-19 hospitalization, we grouped cases into 3 waves, which reflected surges in infections and hospitalizations in the US: wave 1, March through May 2020; wave 2, June through August 2020; and wave 3, September through December 2020.

### Statistical analyses

Descriptive summaries included frequencies and percentages for categorical measures and means with SDs and median values for continuous measures. We used χ^2^ tests to test group differences for categorical variables and *t* tests or ANOVAs (analyses of variance) for continuous measures. We also reported standard mean differences (SMDs), calculated as the absolute difference in means divided by SD, extended to binary and multinomial cases because of large sample sizes (22,23).

We used logistic regression to model associations between readmissions to a VA hospital within 90 days of initial COVID-19 hospital discharge dates and veteran characteristics. Covariates for patient sociodemographic and clinical characteristics and an indicator for COVID-19 wave were included. We checked for interactions between race and insurance coverage and between ventilator use and COVID-19 wave to identify possible issues related to racial disparities and social determinants of health that could be associated with access to care and COVID-19 severity (by using ventilator as a proxy). Because these interactions were not significant at the α = 0.05 level and model coefficients were not qualitatively different, our final model excluded these interactions. Collinearity between variables was assessed descriptively via Pearson correlation or Cramer V where appropriate, and generalized variance inflation factors were used to check for collinearity in the regression models. All analyses were conducted with R version 4.0.2 (R Foundation for Statistical Computing).

## Results

Nationwide, 20,571 veterans were hospitalized in a VA hospital with a COVID-19 positive test from March 1 through December 31, 2020. We excluded 157 admissions with missing data for priority status, admission source, or urban/rural location or that had a date of death before an admission date, yielding 20,414 veterans for analyses. Thirteen percent (n = 2,643) died during their initial COVID-19 hospitalization. Deceased veterans were older (mean, 76.2 y; SD, 10.4 vs 68.1 years, SD, 13.7; SMD, −0.67) and more often male (13.3% vs 5.2%; SMD, 0.18), but did not differ by race or ethnicity. Initial hospital length of stay was longer for the deceased group than for those who survived (13.1 days; SD, 9.3 vs 9.6 days; SD, 11.2; SMD, −0.34), and the deceased group was much more likely to have been on a ventilator during hospitalization (55% vs 7.2%; SMD, 1.20). Veterans who were current smokers, had high comorbidity scores, or were admitted during wave 1 (March–May 2020) were more likely to have died during the initial admission for COVID-19.

For the remaining analyses we examined veterans who were discharged alive from their initial COVID-19 admission (n = 17,771). Sixteen percent (n = 2,764) were readmitted to a VA hospital within 90 days of discharge (Table 1). In unadjusted analyses, veterans readmitted had similar demographic characteristics but had longer initial COVID-19 hospitalizations (14.8 days; SD, 12.7 vs 8.6 days; SD, 10.6; SMD, −0.53) with more patients requiring mechanical ventilation (14.0% vs 6.0%; SMD, 0.26) compared with those not readmitted. More than one-third (38.7%) of those readmitted within 90 days were readmitted for infectious and parasitic diseases, including COVID-19, and 16.1% for diseases of the circulatory system (Table 2). Twenty-two percent of veterans readmitted (n = 598) died within 90 days of their initial hospital discharge compared with 11% of those veterans who were not readmitted.

**Table 1:** Characteristics of Veterans Not Readmitted Versus Readmitted to Hospital Within 90 Days of Discharge Following Initial Hospitalization for COVID-19, March 1–December 31, 2020^a^

Variable	Not readmitted (n = 15,007)	Readmitted within 90 days (n = 2,764)	SMD^b^
**Age, mean (SD)**	67.9 (13.8)	69.3 (13.0)	−0.10
**Sex**			
Male	14,155 (94.0)	2,640 (96.0)	0.05
Female	852 (5.7)	124 (4.5)	
**Race**			
Black	4,477 (30.0)	834 (30.0)	0.02
Other listed races/unknown	9,767 (5.1)	128 (3.6)	
White	763 (65.0)	1,802 (65.0)	
**Ethnicity**			
Hispanic	1,383 (9.2)	238 (8.6)	0.02
Non-Hispanic	13,624 (91.0)	2,526 (91.0)	
**Initial COVID-19 hospital admission length of stay, days, mean (SD)**	8.6 (10.6)	14.8 (12.7)	−0.53
**Ventilator use (yes)**	907 (6.0)	377 (14.0)	0.26
**Body mass index (kg/m^2^), mean (SD)**	30 (7.1)	29.6 (7.0)	0.05
**Smoking status**			
Current	1,640 (11.0)	443 (16.0)	0.24
Former	6,793 (45.0)	1,365 (49.0)	
Never	4,986 (33.0)	793 (29.0)	
Unknown	1,588 (11)	163 (5.9)	
**Marital status**			
Married	6,954 (46.0)	1,240 (45.0)	0.03
Single/other	8,053 (54.0)	1,524 (55.0)	
**Health insurance (fiscal year 2019)**			
VA and private	981 (6.5)	135 (4.9)	0.09
VA and public	3,891 (26.0)	779 (28.0)	
VA only	9,568 (64.0)	1,724 (62.0)	
VA, private and public	567 (3.8)	126 (4.6)	
**VA copay**			
None	8,822 (59.0)	1,584 (57.0)	0.08
Some	4,453 (30.0)	906 (33.0)	
Full copay	1,732 (12.0)	274 (10.0)	
**Discharge location**			
Community	11,507 (77.0)	2,478 (90.0)	0.43
Hospital	343 (2.3)	80 (2.9)	
Long-term care	2,700 (18.0)	92 (3.3)	
Missing	242 (1.6)	34 (1.2)	
Against medical advice	221 (1.5)	82 (3.0)	
**Place of residence**			
Urban	11,061 (74.0)	2,111 (76.0)	0.07
Rural	3,521 (23.0)	598 (22.0)	
Highly rural	425 (2.8)	55 (2.0)	
**Elixhauser (comorbidity) score mean (SD)^c ^**	35.7 (25.8)	48.1 (27.5)	−0.46
**COVID-19 wave**			
1 (March–May 2020)	1,798 (12.0)	293 (11.0)	0.37
2 (June–August 2020)	3,336 (22.0)	561 (20.0)	
3 (September–December 2020)	9,873 (66.0)	1,910 (69.0)	

Abbreviations: SMD, standardized mean difference; VA, Veterans Affairs.

a Values are number (percentage) unless otherwise indicated.

b SMD scores farther from 0 indicate greater difference.

c Range is –4 to 158, Moore et al 2017 (19).

**Table 2:** Readmissions Below and Above the Median Time to Readmission Among Veterans Readmitted to Hospital Within 90 Days of Discharge Following Initial Hospitalization for COVID-19, by Clinical Classification Category, March 1– December 31, 2020^a^

Reason	Total readmissions within 90 days (N = 2,764)	Readmissions within 14 days, (n = 1,362)	Readmissions from 15 to 90 days (n = 1,403)
Certain infectious and parasitic diseases including COVID-19	1,071 (38.7)	742 (54.5)	329 (23.4)
Diseases of the circulatory system	444 (16.1)	158 (11.6)	286 (20.4)
Mental, behavioral, and neurodevelopmental disorders	224 (8.1)	75 (5.5)	149 (10.6)
Diseases of the respiratory system	169 (6.1)	66 (4.8)	103 (7.3)
Symptoms, signs, and abnormal clinical and laboratory findings not elsewhere classified	146 (5.3)	70 (5.1)	76 (5.4)
Diseases of the digestive system	140 (5.1)	52 (3.8)	88 (6.3)
Diseases of the genitourinary system	92 (3.3)	28 (2.1)	64 (4.6)
Injury, poisoning, and certain other consequences of external causes	91 (3.3)	32 (2.3)	59 (4.2)
Diseases of the nervous system	90 (3.3)	37 (2.7)	53 (3.8)
Endocrine, nutritional, and metabolic diseases	87 (3.1)	32 (2.3)	55 (3.9)
Neoplasms	70 (2.5)	29 (2.1)	41 (2.9)
Diseases of musculoskeletal system and connective tissue	41 (1.5)	13 (1)	28 (2.0)
Diseases of skin and subcutaneous tissue	37 (1.3)	11 (0.8)	26 (1.9)
Diseases of blood and blood-forming organs and certain disorders involving the immune mechanism	28 (1.0)	9 (0.7)	19 (1.4)
Factors influencing health status and contact with health services	23 (0.8)	5 (0.4)	18 (1.3)
External causes of morbidity	8 (0.3)	2 (0.1)	6 (0.4)
Diseases of ear and mastoid process	2 (<0.1)	1 (<0.1)	1 (<0.1)
Diseases of eye and adnexa	1 (<0.1)	0	1 (<0.1)

a All data are number (%).

**Figure 1:**
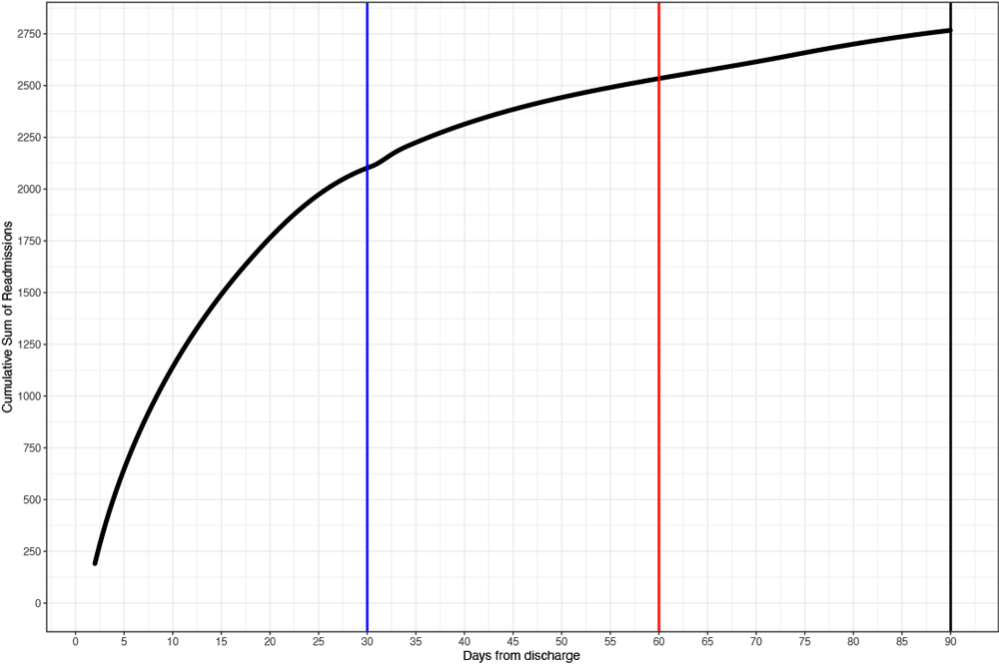
Number and percentage of US veterans (N = 17,771) hospitalized for a diagnosis of COVID-19 who were readmitted to hospital within 90 days of discharge following their initial hospitalization, by number of days between discharge and readmission, March 1–December 31, 2020.

**Figure 2:**
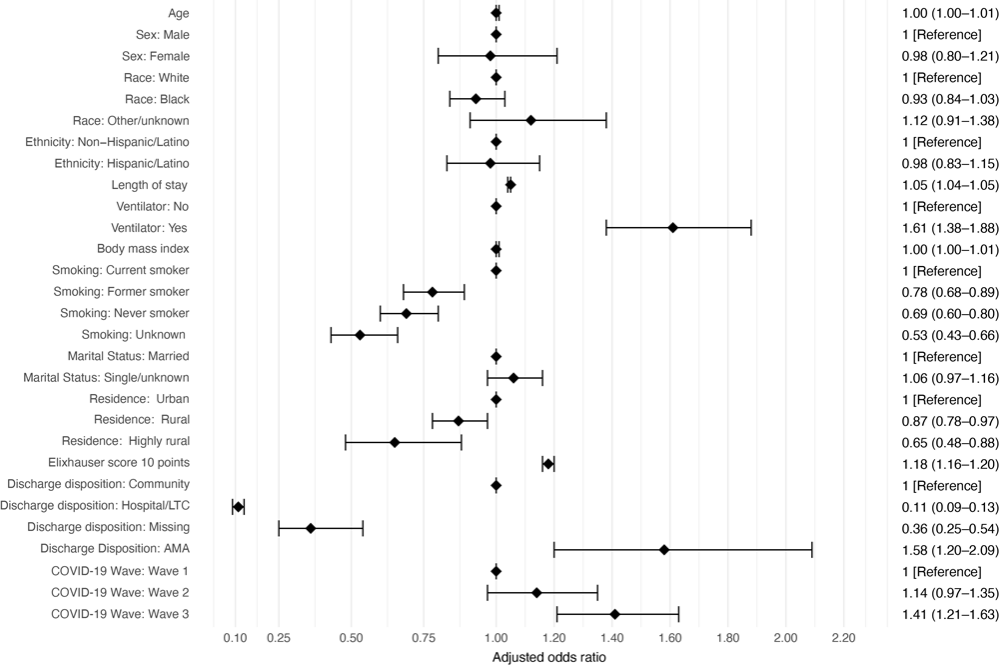
Logistic regression model showing estimated odds of readmission to a Veterans Administration hospital among US veterans (N = 17,771) hospitalized for a diagnosis of COVID-19 who were readmitted to hospital within 90 days of discharge following their initial hospitalization, March 1– December 31, 2020. The Elixhauser score ranges from –0.4 to 15.8 by using the Moore et al method (19) scaled down by 10. Odds ratio and associated 95% CIs for estimated coefficients are represented by points and bars, respectively, and by patient characteristics. Abbreviations: AMA, against medical advice; LTC, long-term care.

Mean time to readmission was 21.6 days (SD = 21.1) with a median of 14 days. Of veterans initially admitted to hospital for COVID-19, 2,097 (11.8%) were readmitted within 30 days, 2,539 (14.3%) within 60 days, and 2,764 (15.6%) within 90 days (Figure 1). Regression analyses identified several variables associated with readmission following COVID-19 discharge. VA hospital readmission within 90 days was associated with initial COVID-19 length of stay, ventilator use, smoking status, urban residence, Elixhauser score, discharge location, and cases in third wave of COVID-19 (Figure 2). A sensitivity analysis excluding veterans with unknown smoking status did not affect the model because differences in the variable coefficients were minimal. When readmission reasons were compared between those occurring within the median of 14 days or greater than 14 days, 54% of readmissions were for infectious and parasitic diseases within 14 days compared with 23% from 15 to 90 days post-discharge.

**Table:** 

Days to readmission	No. readmitted	Cumulative no.	Percentage readmitted
2	191	191	1.08
3	165	356	2.00
4	168	524	2.95
5	117	641	3.61
6	109	750	4.22
7	112	862	4.85
8	92	954	5.37
9	83	1,037	5.84
10	84	1,121	6.31
11	89	1,210	6.81
12	74	1,284	7.23
13	78	1,362	7.66
14	63	1,425	8.02
15	58	1,483	8.35
16	64	1,547	8.71
17	50	1,597	8.99
18	58	1,655	9.31
19	39	1,694	9.53
20	60	1,754	9.87
21	47	1,801	10.14
22	50	1,851	10.42
23	43	1,894	10.66
24	39	1,933	10.88
25	28	1,961	11.03
26	36	1,997	11.24
27	35	2,032	11.43
28	27	2,059	11.59
29	22	2,081	11.71
30	16	2,097	11.80
31	15	2,112	11.88
32	31	2,143	12.06
33	30	2,173	12.23
34	30	2,203	12.40
35	20	2,223	12.51
36	12	2,235	12.58
37	15	2,250	12.66
38	16	2,266	12.75
39	20	2,286	12.86
40	15	2,301	12.95
41	17	2,318	13.04
42	24	2,342	13.18
43	10	2,352	13.24
44	7	2,359	13.27
45	20	2,379	13.39
46	13	2,392	13.46
47	14	2,406	13.53
48	14	2,420	13.61
49	13	2,433	13.69
50	9	2,442	13.74
51	5	2,447	13.77
52	8	2,455	13.82
53	11	2,466	13.88
54	8	2,474	13.92
55	11	2,485	13.98
56	7	2,492	14.02
57	12	2,504	14.09
58	12	2,516	14.16
59	15	2,531	14.24
60	8	2,539	14.29
61	4	2,543	14.31
62	4	2,547	14.33
63	11	2,558	14.39
64	10	2,568	14.45
65	10	2,578	14.51
66	7	2,585	14.55
67	10	2,595	14.60
68	2	2,597	14.61
69	10	2,607	14.67
70	9	2,616	14.72
71	7	2,623	14.76
72	8	2,631	14.81
73	9	2,640	14.86
74	7	2,647	1490
75	7	2,654	14.93
76	10	2,664	14.99
77	13	2,677	15.06
78	10	2,687	15.12
79	10	2,697	15.18
80	8	2,705	15.22
81	4	2,709	15.24
82	7	2,716	15.28
83	9	2,725	15.33
84	9	2,734	15.39
85	8	2,742	15.43
86	5	2,747	15.46
87	6	2,753	15.49
88	2	2,755	15.50
89	6	2,761	15.54
90	3	2,764	15.55

**Table:** 

Patient characteristic	Estimated OR (95% CI)
Age	1.00 (1.00–1.01)
Sex	
Male	Reference
Female	0.98 (0.79–1.2)
Race	
White	Reference
Black	0.93 (0.84–1.03)
Other listed races/unknown	1.12 (0.9–1.38)
Non-Hispanic/Latino	Reference
Hispanic/Latino	0.98 (0.83–1.15)
Length of hospital stay	1.05 (1.04–1.05)
Ventilator	
No	Reference
Yes	1.61 (1.38–1.87)
Body mass index	1 (0.99–1.01)
Smoking status	
Current smoker	Reference
Former smoker	0.78 (0.68–0.89)
Never smoker	0.69 (0.6–0.8)
Unknown	0.53 (0.43–0.65)
Marital status	
Married	Reference
Single or unknown	1.06 (0.97–1.16)
Rurality	
Urban	Reference
Rural	0.87 (0.78–0.97)
Highly rural	0.65 (0.48–0.87)
Elixhauser score 10 points	1.17 (1.16–1.19)
Discharge disposition	
Community	Reference
Hospital or long-term care	0.11 (0.09–0.13)
Missing	0.36 (0.24–0.53)
Against medical advice	1.58 (1.2–2.08)
COVID-19 wave	
Wave 1	Reference
Wave 2	1.14 (0.97–1.35)
Wave 3	1.41 (1.21–1.64)

As the length of initial COVID-19 hospitalizations increased, the odds of being readmitted increased (OR = 1.05; 95% CI, 1.04–1.05; *P* < .001), and veterans who required mechanical ventilation during their initial hospitalization had 1.6 times the odds of being readmitted compared with those who did not require a ventilator (OR = 1.61; 95% CI, 1.38–1.88; *P* < .001). Similarly, veterans with higher comorbidity burdens based on a weighted Elixhauser score had higher odds of being readmitted (OR = 1.18; 95% CI, 1.16–1.20; *P* < .001), and those who were former smokers, never smoked, or had a missing smoking status were less likely to be readmitted compared with current smokers (former smokers: OR = 0.78; 95% CI, 0.68– 0.89; never smoked, OR = 0.69; 95% CI, 0.60–0.80; smoking status missing, OR = 0.53; 95% CI, 0.43–0.66; all *P* <.001). In addition, veterans who lived in rural or highly rural areas had lower odds of being readmitted than those living in urban areas (rural area: OR = 0.87; 95% CI, 0.78–0.97; *P* = .01; highly rural area: OR = 0.65; 95% CI, 0.48–0.88; *P* = .005). Odds of readmission were greater during the third wave of COVID-19 compared with the first wave (OR = 1.41; 95% CI, 1.21–1.63; *P* < .001). Examination of patient characteristics and outcomes across the 3 waves found that the average length of initial hospital stay decreased from 13 days (SD, 14.8) to 10.4 days (SD, 11.7) to 8.7 days (SD, 10.0) by wave 3 (Table 3).

**Table 3:** Patient Characteristics by COVID-19 Wave, Veterans Readmitted to Hospital Within 90 Days of Discharge Following Initial Hospitalization for COVID-19, March 1– December 31, 2020

Characteristic	COVID-19 Wave (2020)		
	1: 3/1–5/31 (N = 2,021)	2: 6/1–8/31 (N = 3,897)	3: 9/1–12/31 (N=11,783)
Age, mean (SD), y	67.9 (13.9)	66.2 (14.4)	68.7 (13.4)
Male, %	94.8	93.5	94.8
White, %	47.8	58.6	70.3
Initial COVID-19 hospital length of stay, mean (SD)	13.0 (14.8)	10.4 (11.7)	8.7 (10.0)
Ventilator use, %	11.7	7.6	6.3
Current smoker, %	12.2	11.9	11.6
Discharge to community, %	69.7	78.0	80.5
Discharged against medical advice, %	1.0	1.9	1.8
Readmitted within 90 days, %	14.0	14.4	16.2
Death within 90 days, %	14.4	12.1	11.9
Rural/highly rural place of residence, %	10.9	22.1	29.8
Elixhauser score, mean (SD)^a^	40.3 (27.4)	37.6 (27)	37.2 (26)

a Range –4 to 158, Moore et al 2017 (19).

## Discussion

Veterans who were readmitted had longer initial COVID-19 hospitalizations and were also more likely to have been on mechanical ventilation, be current smokers, and live in urban areas. Veterans who were readmitted also had more comorbidities than those not readmitted, suggesting that COVID-19 may have exacerbated existing conditions, triggering readmission. More than 60% of readmissions were not due to an infectious disease, including COVID-19. Of note, characteristics of the infected cohort shifted over time; during the first wave, hospitalized veterans were more often Black and were more likely to die during initial hospitalization compared with the third wave, which involved a much larger number of veterans admitted, more White veterans, and more veterans who lived in rural areas. The third wave included the largest number of people infected up to that point in the pandemic. Yet we observed that the length of the initial COVID-19 hospital stay decreased over time. We do not know whether the decrease in length of stay was due to patients recovering more quickly than in earlier waves, improvements in care due to increased physician experience treating COVID-19 patients, or both or to a shift in practice to discharge patients sooner to accommodate the increased demand for hospital beds and a fixed bed capacity. Hospitals were overwhelmed with admissions in late 2020 with a large surge of infections, and veterans possibly were discharged earlier than normal because of a shortage of beds.

Very little has been published about hospital capacity during the COVID-19 surges. Numerous news articles were published about how overburdened hospitals had to convert general beds to ICU beds and keep COVID-19 patients in general beds when the ICU was at capacity. The demand for nursing home beds also affected hospital bed capacity, particularly in the first year of the pandemic. In the peer-reviewed literature, one article by Bravata et al examined the association between ICU patient load and demand and mortality rates in US VA hospitals from March through November 2020 (closely matching the time frame of our study) (24). They found that patients who were treated in ICUs during periods of high COVID-19 ICU demand were at increased risk of death. The proportion of COVID-19 patients treated in general wards also increased over this period. The authors hypothesized that patients with COVID-19 who would have been admitted to the ICU under normal circumstances were admitted to the wards. In such cases, COVID-19 patients who did not require ICU-level care may have been discharged earlier from general wards to address the increased bed demand, particularly bed demand for very sick patients. Examination of turnover in non-ICU hospital beds during this period warrants further study.

In the first 10 months of the COVID-19 pandemic, more than 20,000 veterans were hospitalized in VA hospitals with a diagnosis of COVID-19; 13% died in the hospital. Our observed death rate during initial hospitalization is lower than reported in other studies (8,26,27). Sixteen percent of veterans discharged alive were readmitted within 90 days. It is difficult to compare readmission rates, however, because each study used a different time point to report readmissions, ranging from 2 weeks to 30 or 60 days. Readmission results to date suggest that time to readmission following COVID-19 discharge tends to be short (less than 2 weeks on average). Donnelly et al (3) used a cohort of hospitalized veterans from early in the pandemic and reported a 60-day readmission rate of 20%. Our sample of veterans was younger than in Donnelly (68.1 y vs 70.2 y), 10 times larger, covered a longer period, and focused on readmission to acute care, likely accounting for the differences observed in the readmission rates between studies (12). Furthermore, more than half of readmissions within 14 days were due to infectious diseases, suggesting that COVID-19 symptoms prompted readmission. Readmissions that occurred from 15 to 90 days were for various potential chronic conditions of the circulatory, respiratory, digestive, and endocrine systems.

Although it could be argued that patients discharged to hospice or long-term care are more likely to die soon after discharge than those discharged to the community, we did not find this to be the case. A sensitivity analysis we conducted that excluded the 3,213 people admitted to institutional care settings resulted in very little change in our model. Eighteen percent of those discharged to the community were readmitted within 90 days, driven by increasing surges of infections, ventilator use during the first admission, urban residence, White race, and current smoking status.

The data presented here represent initial infections that occurred before the availability of vaccinations for COVID-19. Vaccines were made available to the public beginning in January 2021 and were first offered to people aged 65 years or older and those at highest risk. We did not have data on whether readmissions may have been triggered by re-infection, because this would have required evidence of a negative test following the initial COVID-19 infection, and then a new positive test. Our data do suggest that early readmissions were likely related to COVID-19. Data from Germany looking at follow-up 6 months after initial COVID-19 hospitalization reported that 13% of their readmissions were due to people still being infected with COVID-19 or being re-infected, suggesting that either reason could trigger readmission (9).

Although COVID-19 infections have continued through 2021 and into 2022, much of the literature has shifted focus to the post-acute sequalae of COVID-19. A significant proportion of people infected with COVID-19 have experienced symptoms beyond 12 weeks of the initial infection (28). Symptoms occurring this far out from infection include extreme fatigue, shortness of breath, brain fog, joint pain, anxiety, and depression. Although these symptoms are unlikely to result in hospitalization, they are debilitating and have a substantial effect on quality of life (28). Recent data suggest that contracting COVID-19 after being vaccinated not only reduces the likelihood of hospitalization and severe infection (29), but also may be associated with decreased likelihood of readmission and decreased prevalence of post-acute sequalae of COVID-19 infection (30). More research is needed in this area. Continuing shifts in variants of COVID-19 along with availability and effectiveness of vaccines and newer treatments, such as antiviral therapy, point to the importance of monitoring changes in patient outcomes.

Our study had limitations. We relied on electronic medical records data and were limited to data captured there. Also, some relevant data may have been embedded within clinical notes, requiring additional effort to extract and standardize. The electronic medical record was also a strength of this study because it allowed us to account for the hospitalization and readmission experience of a large, national cohort. Results were based on initial infections that occurred before the availability of vaccines, which have had a substantial impact on outcomes. Finally, we limited our study to veterans treated at VA hospitals, so results may not be generalizable to those treated in other health systems. Additional research is needed to collect data on veterans treated outside the VA system.

The ongoing shifting of the COVID-19 virus along with the availability of vaccines and vaccine boosters has influenced the outcomes for people infected with the virus. Readmissions for possible COVID-19–related issues within a short time of initial discharge in a subset of our cohort point to the need to continue to monitor outcomes and to identify interventions to mitigate these negative consequences.
